# Coronary Microvascular Dysfunction: Features and Prognostic Value

**DOI:** 10.3390/jcm12082964

**Published:** 2023-04-19

**Authors:** Kristina Kopeva, Elena Grakova, Alina Maltseva, Andrew Mochula, Anna Gusakova, Andrew Smorgon, Konstantin Zavadovsky

**Affiliations:** 1 Department of Myocardial Pathology, Cardiology Research Institute, Tomsk National Research Medical Center, Russian Academy of Sciences, Tomsk 634009, Russia; gev@cardio-tomsk.ru; 2 Nuclear Department, Cardiology Research Institute, Tomsk National Research Medical Center, Russian Academy of Sciences, Tomsk 634009, Russia; man@cardio-tomsk.ru (A.M.); smu@cardio-tomsk.ru (A.M.);; 3 Department of Laboratory and Functional Diagnostics, Cardiology Research Institute, Tomsk National Research Medical Center, Russian Academy of Sciences, Tomsk 634009, Russia; anna@cardio-tomsk.ru (A.G.); sav@cardio-tomsk.ru (A.S.)

**Keywords:** coronary microvascular dysfunction, non-obstructive coronary artery disease, biomarkers, inflammation, fibrosis, preserved ejection fraction

## Abstract

(1) Background: The results of the international studies support the assumption that coronary microvascular dysfunction (CMD) occurs significantly more often than previously identified and is associated with adverse outcomes. However, there is a lack of the accurate comprehension of its pathophysiology. The objectives of this study were to evaluate the clinical and instrumental features of CMD and to assess its prognostic value during 12 months of follow-up period. (2) Methods: A total of 118 patients with non-obstructive coronary artery disease (CAD) and preserved LV ejection fraction (62 [59; 64]%) were enrolled in the study. Serum levels of biomarkers were analyzed by enzyme-linked immunoassay. CMD was defined as the reduced myocardial flow reserve (MFR) ≤ 2 obtained by dynamic CZT-SPECT. Two-dimensional transthoracic echocardiography with evaluation of LV diastolic dysfunction was performed baseline. (3) Results: Patients were divided into groups depending on the presence of CMD: CMD+ group (MFR ≤ 2; *n* = 45), and CMD− group (MFR > 2; *n* = 73). In CMD+ group, the severity of diastolic dysfunction, the levels of biomarkers of fibrosis and inflammation were higher than in CMD− group. Multivariate regression analysis showed that the presence of diastolic dysfunction (OR 3.27; 95% CI 2.26–5.64; *p* < 0.001), the hyperexpression of NT-proBNP ≥ 760.5 pg/mL (OR 1.67; 95% CI 1.12–4.15; *p* = 0.021) and soluble ST2 ≥ 31.4 ng/mL (OR 1.37; 95% 1.08–2.98; *p* = 0.015) were independent factors associated with CMD. Kaplan–Meier analysis showed that a rate of the adverse outcomes was significantly (*p* < 0.001) higher in patients with CMD (45.2%, *n* = 19) than in patients without it (8.6%, *n* = 6). (4) Conclusions: Our data suggest that the presence of CMD was associated with the severe diastolic dysfunction and hyperexpression of the biomarkers of fibrosis and inflammation. Patients with CMD had higher rate of the adverse outcomes than those without it.

## 1. Introduction

The microvascular structures in the heart play the crucial role in regulation of systemic hemodynamics, tissue oxygenation and nutrition, transport of mediators, exchanges of gases and metabolites to and from tissues [1]. A central mechanism that efficiently accommodates these functions is vasomotion defined as rhythmic changes in arteriolar diameter, and results from integration of local tissue (e.g., CO_2_, adenosine), circulating (e.g., hormones, cytokines) and neurogenic signals. Normal microvascular function is an optimal performance of the arterioles, capillaries and venules to adjust their respective functions [2]. However, some structural and functional abnormalities of coronary microvasculature occur due to dyslipidemia, hypertension, smoking, diabetes, overweight and obesity, physical inactivity, unhealthy diet, older age, etc. [3]. Coronary microvascular dysfunction (CMD) is defined as a type of non-obstructive coronary artery disease (CAD) that causes the small blood vessels feeding the heart muscle to not work as they should, and it has been coined to refer to this clinical condition and is increasingly recognized as an important clinical entity in many clinical settings [3]. In 1967 Likoff et al. first suggested that CMD could play an important role in patients with non-obstructive CAD and be a crucial contributor to heart failure with preserved ejection fraction (HFpEF) [4]. Later, in the study with the endomyocardial biopsy samples, patients with HFpEF were revealed to have lower microvascular density and more myocardial fibrosis than healthy controls [5].

The results of the international trials using invasive or non-invasive diagnostic methods support the assumption that CMD occurs significantly more often than previously identified, especially in patients with HFpEF. Murthy et al. reported that 53% of patients with non-obstructive CAD who present with chest pain had evidence of inducible myocardial ischemia [6]. According to the latest meta-analysis data of 56 studies comprising 14,427 patients, the proportion of patients with CMD was 41% in general population [7]. Moreover, the incidence of CMD increased up to 75–85% when its prevalence was analyzed in patients with HFpEF [8,9,10]. Thereby, a new paradigm, in which CMD is at the center of myocardial ischemia and HFpEF, has been gaining support over the last years. 

Several observations have shown that CMD is associated with significant increasing in major adverse cardiovascular events (MACE) [9,11,12,13,14]. Moreover, despite compelling evidence in support of microvascular disturbances as a predictor of adverse outcomes, the potential mechanisms of CMD development are not still understand and appear to be heterogeneous, encompassing cell metabolic disorders, inflammation, reactive oxygen species formation, enhanced coronary vasoconstrictive reactivity, impaired endothelium-dependent and independent vasodilator capacities, hormonal and electrolyte imbalance, etc., and, eventually, the development of fibrosis and increased stiffness, and the exacerbation of coronary microvascular resistance secondary to structural factors [12,13]. 

However, nowadays there is still a lack of comprehensive characteristics of CMD. Thus, the objectives of this study were to evaluate the clinical and instrumental features of CMD in patients with non-obstructive CAD and preserved LV ejection fraction (LVEF) and to assess the prognostic value of CMD during 12 months of follow-up period. 

## 2. Materials and Methods

### 2.1. Study Population

From January 2020 to January 2021, a total of 118 low-risk patients (70 men, median age of 62.0 [58.0; 69.0] years) with non-obstructive CAD and preserved LVEF (62 [59; 64]%) were enrolled in the study.

Inclusion criteria: (1) non-obstructive (<50%) CAD confirmed by coronary computed tomography angiography; (2) documented LVEF ≥ 50% measured by echocardiography; (3) sinus rhythm; (4) capability of giving signed informed consent. 

Exclusion criteria: (1) previous myocardial infarction; (2) planned coronary revascularization and/or previous revascularization of coronary arteries; (3) systolic blood pressure > 160 or <90 mmHg; (4) atrioventricular block II-III degree and/or sinus node weakness syndrome; (5) persistent or permanent atrial fibrillation and/or flutter; (6) moderate or severe stenosis and/or insufficiency of the heart valves; (7) hypertrophic and dilated cardiomyopathy; (8) prior pulmonary embolism with pulmonary hypertension ≥ 45 mmHg; (9) severe bronchial asthma and/or chronic obstructive pulmonary disease; (10) thyroid gland disorders; (11) reduced glomerular filtration rate (CKD-EPI) < 30 mL/min/m^2^; (12) hepatic insufficiency classified as Child–Pugh C; (13) acute and chronic inflammatory heart disease; (14) hemoglobin level < 100 g/dL; (15) cerebrovascular events within 90 days prior to enrollment; (16) body mass index > 35 kg/m^2^; (17) ventricular extrasystole ≥ 3 grades (Lown).

### 2.2. Dynamic Single-Photon Emission Computed Tomography (SPECT)

Patients were instructed to refuse caffeinated foods or beverages and methylxanthine-containing substance use for at least 24 h before the procedure and to avoid taking nitrates, calcium channel blockers and beta-blockers for at least 48 h before the procedure. All scans were performed after overnight fasting and with a sinus heart rhythm [15]. The protocol of the study, data acquisition and analysis were described in the previous articles [15,16]. Briefly, a rest-stress two-day protocol was carried out. Before the first dynamic acquisition, a low-dose computed tomography scan (tube voltage 100 kV, tube current 20 mA, rotation time 0.8 s, helical pitch 0.969:1, slice thickness 5 mm and interstice interval on 5 mm) had been performed for heart positioning.

During the study at rest 3 MBq·kg^−1^ of 99mTc-Sestamibi were injected using a syringe pump intravenously as a 5 mL bolus (injection rate 1 mL·s^−1^) followed by saline flush (30 mL with the rate 2 mL·s^−1^). List mode electrocardiogram-gated dynamic data acquisition started just before the radiopharmaceutical bolus injection and was acquired for 610 s. 

Pharmacological stress-test with adenosine (intravenous dose of 160 mcg·kg^−1^·min^−1^) was provided of standard 4-min protocol [17]. Within 2 min of intravenous adenosine infusion, a dose of 99mTc-Sestamibi (3 MBq·kg^−1^) was injected and list mode dynamic data acquisition of 610 s was started just before the radiotracer injection. The infusion of adenosine continued for another 2 min. 

Both days after 60 min from tracer injection, the 7-min-long-standard electrocardiogram-gated (16 framed per cardiac cycle) rest/stress acquisition was performed. All studies were analyzed on the dedicated workstation Corridor 4DM SPECT и 4DM Reserve v. 2015 (INVIA, Ann Arbor, MI, USA). Raw dynamic SPECT image was transformed into 20 frames: 12 frames of 10 s and 8 frames of 30 s. After, the time–activity curves for the input function, LV myocardium and the left anterior descending artery, left circumflex artery and right coronary artery territories were automatically generated. The myocardial retention rate was estimated using generalized net retention model. 

The net tracer retention model was applied to calculate the retention rate *R* using the following equation.

$$R=\frac{\frac{1}{PV}\cdot \frac{1}{\left(t_{3}-t_{2}\right)}\int_{t_{2}}^{t_{3}}C_{m}\left(t\right)-S_{m}\cdot C_{a}\left(t\right)dt}{CF\int_{0}^{t_{1}}C_{a}\left(t\right)-S_{b}\cdot C_{m}\left(t\right)dt}$$

where *R* denotes the retention rate, *C_m_*(*t*) and *C_a_*(*t*) denotes the activity concentrations in the myocardium and blood respectively; *t*_1_ denotes the end of the blood-pool phase, *t*_2_ and *t*_3_ denotes the start and finish times of the interval used to measure myocardial uptake. *PV* denotes partial volume correction; *CF* denotes correction factor for myocardial density; *S_m_* and *S_b_* denotes the spill-over constants from blood-to-myocardium and myocardium-to-blood, respectively.

In order to convert the tracer retention rate to MBF values the Renkin–Crone flow model was used with parameters α = 0.880, β = 0.208 [18]. As a result, quantitative indices (stress-MBF and rest-MBF) were assessed. The value of MFR was calculated as stress-MBF/rest-MBF ratio. Additionally, motion correction and attenuation correction were used [16,17]. In the absence of overt CAD reduced MFR ≤ 2 was defined as a marker of CMD [3]. 

Myocardial perfusion imaging analysis was performed as follows: each of 17 segments was scored based on semiquantitative 5-point scoring system (from 0 = normal uptake to 4 = absent radiotracer distribution). Accordingly, the sum of the stress scores of all segments (SSS) and the sum of the rest scores of all segments (SRS) were quantified. The summed difference score (SDS) was calculated as the difference between SSS and SRS [17].

### 2.3. Echocardiography

Philips Affiniti 70-ultrasound scanner with advanced imaging was used to perform two-dimensional transthoracic echocardiography. All studies were performed by one high-quality specialist that excluded a high intra- or inter observer variability. LV myocardial mass index (LVMMI) and left atrial volume index (LAVI) were calculated using the following equations. Left ventricle mass was calculated using the Devereux formula: 0.80 × {1.04 × [(septal thickness + internal diameter + posterior wall thickness)3 − (internal diameter)3]} + 0.6 g. The left ventricular mass was indexed according to body surface area: LVMMI = left ventricular mass/body surface area. Left atrial volume determination was performed using the biplane Simpson’s method. The left atrial volume was indexed according to body surface area: LAVI = left atrial volume/body surface area. Evaluation of left ventricle diastolic dysfunction was based on six indices: E wave, E/A ratio, septal or lateral e′, average E/e′, left atrial volume indexed and the tricuspid regurgitation peak velocity. Diastolic dysfunction was diagnosed in the presence of ≥3 abnormal values [18]. LV global longitudinal strain (GLS) by speckle tracking echocardiography was measured manually in 17-segments LV model as the average segmental value based on three apical imaging planes [19]. GLS was analyzed using the commercial software for speckle tracking analysis (GE Echo PAC v.112.1.7, GE Healthcare). GLS was calculated and expressed as an average of all analyzed segmental strain values. Strain values were presented in absolute numbers. According to the recommendations, segments with unacceptably poor tracking quality were eliminated from the analysis [20].

### 2.4. Blood Sampling and Biochemical Analysis

Blood samples were obtained by venipuncture, and adequate centrifuged serum samples were stored at −26 °C with a single freeze–thaw cycle. Serum levels of biomarkers were analyzed by enzyme-linked immunoassay (NT-proBNP, Biomedica immunoassays, Vienna, Austria; Fibroblast growth factor 23, Biomedica immunoassays, Austria; Tissue inhibitor of metalloproteinase-1, Biomedica immunoassays, Austria; soluble ST2, Presage^®^ Analysis ST2, Critical Diagnostics, USA; Matrix metalloproteinase 9, eBioscience, San Diego, CA, USA; tetranectin, eBioscience, USA; interleukines-1β, 6 and 10; Vector-Best, Russia; high-sensitivity C-reactive protein, Biomedica immunoassays, Austria).

### 2.5. Study Outcomes

The adverse outcomes were defined as time to the as new or worsening symptoms/signs of HFpEF, hospitalization due to decompensation of HFpEF or cardiac death.

New onset of HFpEF was diagnosed in accordance with the 2021 ESC guidelines for the diagnosis and treatment of acute and chronic heart failure as symptoms and signs of HF, with evidence of structural and/or functional cardiac abnormalities and/or raised natriuretic peptides, and with an LVEF ≥ 50% [21].

### 2.6. Statistical Analysis

All analyses were performed with STATISTICA V10.0 software package or R software, version 2. The sample data were not normally distributed; therefore, nonparametric analyzes were used. Categorical variables are expressed as counts and percentages. Continuous variables are expressed as the median (25th–75th percentiles (P25–75). Rank correlation was analyzed by Spearman’s rank correlation coefficient. Statistical differences between two groups were compared using the χ2 test for categorical variables and using Mann–Whitney U test for continuous variables. The cutoff points for continuous predictors were found by ROC-analysis. Multivariate logistic regression analysis was used to assess the impact of baseline clinical parameters and imaging variables on the risk of CMD. Baseline variables that did not have a high degree of collinearity (r ≥ 0.7) considered to be clinically relevant or to have a univariate relationship with CMD (*p* < 0.05) were entered as covariates in a multiple logistic regression model: age, sex, heart rate, mean arterial pressure, hemoglobin, serum sodium, serum potassium, estimated glomerular filtration rate, lipids, biomarkers, medication prescribed for heart failure, comorbidities, echocardiographic and dynamic SPECT parameters. All *p*-values are two-tailed. In all statistical analyses, a two-tailed *p*-value of 0.05 or less was considered to indicate statistical significance.

## 3. Results

Patients were divided into groups depending on the presence of CMD: CMD+ group (MFR ≤ 2; *n* = 45), and CMD− group (MFR > 2; *n* = 73). Initially, HFpEF of NYHA I-III functional classes was diagnosed in 58 patients (49.2%) in accordance with the 2021 ESC guidelines for the diagnosis and treatment of acute and chronic heart failure [21]. 

### 3.1. Baseline Clinical and Demographic Characteristics

Patients with CMD were more likely to be diagnosed with HFpEF (*p* < 0.001), had a history of type 2 diabetes mellitus (*p* = 0.007) and were more often smokers (*p* = 0.009) than patients without CMD. Other baseline clinical and demographic characteristics of patients did not differ between groups. Since the diagnosis of HFpEF was established for the first time, at the time of inclusion in the study, patients did not receive optimal medical therapy: the frequency of prescribing β-blockers was 15.3%, angiotensin-converting enzyme inhibitors/angiotensin II receptor antagonists—11.9%, statins—19.5%, diuretics—14.4% and antiarrhythmic drugs—5.6%. The groups did not differ significantly in the frequency of distribution of prescribed drugs (Table 1). Subsequently, the treatment was corrected and optimal medical therapy was prescribed according to current clinical recommendations. 

### 3.2. Echocardiographic and Dynamic SPECT Parametrs

In patients with CMD, the severity of diastolic dysfunction was higher than in patients without it. The lateral e′ values were lower by 35% in CMD+ group (*p* = 0.009) than in CMD− group. The peak rate of tricuspid regurgitation was higher by 12% (*p* = 0.011), the E/e′ ratio was higher by 21.4% (*p* = 0.041) and LAVI by 51.2% (*p* = 0.038) in CMD+ group than CMD− group. In patients with CMD, the absolute value of GLS was lower by 29.7% (*p* = 0.005) than in those without it. Other echocardiographic parameters did not differ significantly between groups (Table 2). In patients with CMD, MFR values were lower by 48.3% (*p* < 0.001) than in patients without it. In CMD+ group, rest-MBF was higher by 30.1% (*p* < 0.001) and stress-MBF was lower by 30.1% (*p* < 0.001) compared to CMD− group. Standard semi-quantitative indices of myocardial perfusion imaging were found to be negligible between groups (Table 2). 

### 3.3. The Levels of Biomarkers

Despite the values of hsCRP and interleukins did not exceed the reference intervals, the differences were found in their levels depending on the CFR values. Thus, hsCRP concentrations were higher by 1.8 times (*p* = 0.011) in CMD+ group compared to CMD− group. Interleukin-6 levels did not differ significantly between groups (*p* = 0.842), while interleukin-10 concentrations were lower by 21.7% (*p* = 0.048) in patients with CMD than in those without it, and interleukin-1β was 2.7-fold higher (*p* = 0.046) in CMD+ group compared to CMD− group. The levels of NT-proBNP were 2.6-hold greater (*p* = 0.004), soluble ST2 concentrations were higher by 18.1% (*p* < 0.001), TIMP-1 levels were 2.3-hold higher (*p* = 0.011) and MMP-9 levels were 1.9-hold greater (*p* = 0.012) in CMD+ group compared to CMD− group (Table 3). GDF-23 and tetranectin did not differ between groups.

### 3.4. Diagnostic Value

Univariate regression analysis revealed that the presence of type 2 diabetes mellitus (OR 1.43; 95% CI 1.17–3.57; *p* = 0.012), diastolic dysfunction (OR 3.18; 95% CI 1.16–4.12; *p* < 0.001), smoking (OR 2.01; 95% CI 0.99–2.43; *p* = 0.043), the hyperexpression of NT-proBNP ≥ 760.5 pg/mL (OR 2.13; 95% CI 1.78–4.87; *p* = 0.009), high-sensitivity C-reactive protein ≥ 2.7 g/L (OR 1.63; 95% CI 0.98–2.54; *p* = 0.013) and soluble ST2 ≥ 31.4 ng/mL (OR 1.97; 95% 1.16–5.12; *p* = 0.003) were associated with CMD (Table 4). Hence, multivariate regression analysis showed that the presence of diastolic dysfunction (OR 3.27; 95% CI 2.26–5.64; *p* < 0.001), the hyperexpression of NT-proBNP ≥ 760.5 pg/mL (OR 1.67; 95% CI 1.12–4.15; *p* = 0.021) and soluble ST2 ≥ 31.4 ng/mL (OR 1.37; 95% 1.08–2.98; *p* = 0.015) were independent factors associated with CMD. Other baseline clinical and imaging parameters did not show significant value. 

### 3.5. Correlative Links

MFR and rest-MBF levels correlated with NT-proBNP levels (r = −0.368; *p* = 0.007 and r = 0.354; *p* = 0.042, respectively). MFR values also correlated with soluble ST2 (r = −0.5674; *p* = 0.001), LAVI (r = −0.464; *p* = 0.001), septal e′ (r = 0.314, *p* = 0.012) and GLS (r = 0.723, *p* < 0.001). Rest-MBF correlated with E/e′ (r = 0.512; *p*= 0.002). Interleukin-10 levels correlated with MFR (r = 0.511, *p* = 0.005), rest-MBF (r = −0.432, *p* = 0.045) and stress-MBF (r = 0.317; *p* = 0.012); interleukin levels 1β significantly correlated with MFR (r = −0.371; *p* = 0.046) and E/e′ (r = 0.278; *p* = 0.019) values, and hsCRP concentrations correlated with MFR values (r = −0.412; *p* = 0.019).

### 3.6. Prognostic Value

**Figure 1:**
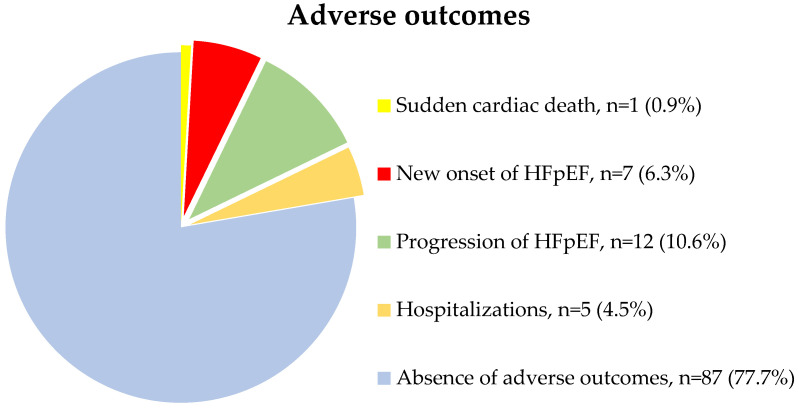
The adverse outcomes registered during 12 months of follow-up period.

Six patients withdrew from the study due to loss of contact with them. During the 12 months of follow-up, 25 patients had the adverse outcomes. Sudden cardiac death was registered in one (0.9%) case; new onset of HFpEF was diagnosed in 7 (6.3%) patients; progression of heart failure by 1 or more NYHA functional classes was detected in 12 (10.6%) patients, of which one (0.9%) patient required outpatient administration of IV diuretic therapy with its subsequent intensification per os, the rest 11 patients had just increasing the dosage of oral diuretic therapy; 5 (4.5%) patients required hospitalization due to decompensated HFpEF (Figure 1).

**Figure 2:**
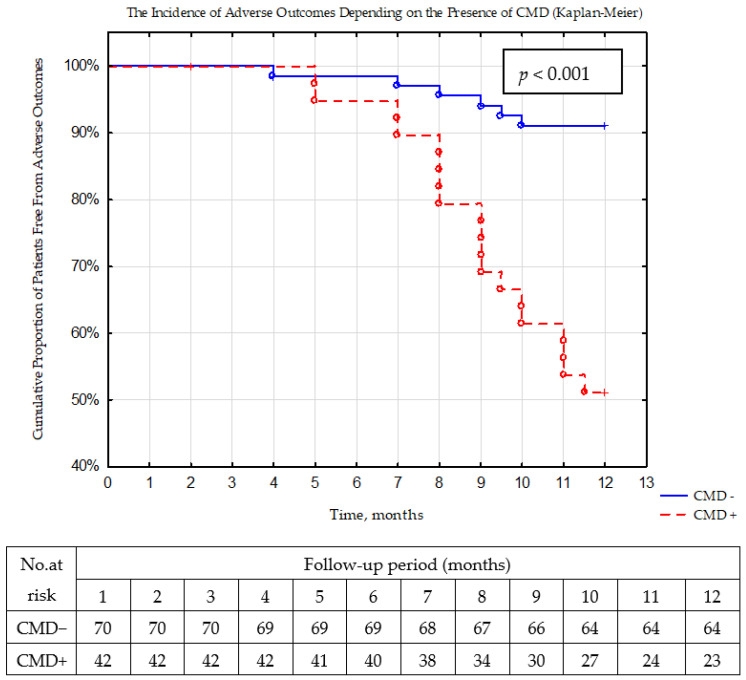
The rate of adverse outcomes during 12 months of follow-up period depending on the presence of coronary microvascular dysfunction (Kaplan–Meier).

Based on Kaplan–Meier analysis, the rate of the adverse outcomes significantly (*p* < 0.001) differed between groups (Figure 2). The incidence of adverse events preponderated (45.2%, *n* = 19) in patients with CMD, than in patients without it (8.6%, *n* = 6).

## 4. Discussion

Our results support the notion that CMD obtained with dynamic CZT imaging is associated with severe diastolic dysfunction, subtle LV systolic function impairment and is related to overexpression of the biomarkers of fibrosis and inflammation in low-risk patients with non-obstructive CAD and preserved LVEF. Presence of CMD in this population is interrelated with greater rate of adverse events and can individuate patients at high risk of adverse outcomes during the 12-month follow-up period. These results could open the door to the development of preventive and therapeutic strategies that could improve the quality of life of affected patients and prognosis.

Since Paulus and Tschöpe postulated a central role of CMD in the etiology of myocardial ischemia, a substantial number of the studies have investigated the role of CMD in cardiovascular diseases [22]. Several observations have shown that CMD could be of considerable importance of myocardial damage, possibly, because impaired myocardial perfusion causes ischemia and cardiomyocyte injury, leading to a depressed cardiac functional reserve and the development of myocardial fibrosis [12,14,22,23,24,25]. 

Whilst the results of the COURAGE and ISCHEMIA studies have shown that coronary revascularization is not associated with significant reduction in MACE, the accumulated evidence supports the hypothesis that MACE risk reduction is targeted beyond epicardial CAD [26,27]. The first international COVADIS study has provided novel evidence that CMD is an important health problem and it portends a substantial risk for MACE [28]. Later it was established that across a broad range of pathologies and patient cohorts, CMD was associated with an increased hazard of all-cause mortality and MACE [10]. Schroder J. et al. showed that reduced CMD assessed by Doppler echocardiography in the left anterior descending artery as coronary flow velocity reserve was also interdependent on the risk of repeated hospital admission for angina and all-cause mortality [29]. Kato S. et al. obtained and analyzed cardiovascular magnetic resonance-derived MFR data from 163 HFpEF patients (73 ± 9 years; 86 [53%] female). MFR was significantly lower in HFpEF with adverse events compared with those without it (1.93 ± 0.38 vs. 2.67 ± 0.52, *p* < 0.001) and was a predictor for cardiovascular death and HF hospitalization [30]. In our study, the incidence of adverse events also prevailed in patients with reduced MFR than in those with preserved one. At the same time, only adverse events associated with the progression and development of HFpEF were registered during 12 months of observation, which confirms the fact that the presence of CMD in patients with non-obstructive CAD is primarily a driver of HFpEF. 

We also demonstrated that dynamic CZT-SPECT quantifications were associated with the biomarkers of fibrosis and inflammation: MFR and rest-MBF correlated with NT-proBNP concentrations, MFR correlated with soluble ST2, interleukin-10, interleukin 1β and hsCRP concentrations. All these findings have supported the interconnection between chronic inflammation, perivascular fibrosis and CMD. The systemic inflammatory condition caused by comorbidities such as type 2 diabetes mellitus, obesity and chronic obstructive pulmonary disease can lead to increased production of endothelial reactive oxygen species. The reaction between reactive oxygen species cofactors and eNOS reduces the production and bioavailability of nitric oxide. As a result, reduced availability of nitric oxide reduces the level of protein kinase G, which is required for phosphorylation of titin, a cytoskeletal protein responsible for diastolic function and myocardial extensibility [14]. Therefore, impaired titin phosphorylation due to dysregulation of the nitric oxide-protein kinase G axis contributes to a decrease in diastolic reserve. It was shown that patients with CMD had greater microvascular rarefaction and more pronounced myocardial fibrosis, as well as a lower microvascular density compared to the control group [12]. Moreover, microvascular endothelial inflammation could be a possible trigger for the development of CMD and myocardial fibrosis. In our study, patients with CMD more often had a history of type 2 diabetes mellitus (*p* = 0.003) and were smokers (*p* = 0.012). This once again confirms the contribution of comorbid pathology to the formation of CMD [31]. In a large crossover study including patients with type 2 diabetes and risk factors for heart failure (*n* = 336), the prevalence of CMD (MFR < 2.5 according to Doppler echocardiography) was 59% [32]. Additionally, 76 patients underwent phenotyping for a range of markers of endothelial dysfunction, inflammation and diastolic dysfunction. Seventeen inflammatory biomarkers were negatively correlated with MFR and 15 were positively correlated with E/e′. Both CFR and E/e′ correlated only in the subgroup of patients with CMD and signs of elevated filling pressure (E/e′ > 10; *p* = 0.012) [32]. 

Ahmad A. showed that CMD was inversely associated with filling pressures, particularly during exercise [24]. In another study including suspected CAD patients with preserved LVEF who underwent positron emission tomography (PET), impairment of PET-derived MFR was associated with diastolic dysfunction and future development of HFpEF hospitalization [33]. Thus, patients with impaired MFR obtained by PET demonstrated linearly decreasing e′ and increasing E/e′ consistent with worsening diastolic function (*p* < 0.0001). In adjusted analyses, impaired MFR was independently associated with diastolic dysfunction (E/e′septal > 15, adjusted OR 2.58, 95% CI 1.22–5.48) and composite cardiovascular outcomes or HFpEF hospitalization alone (adjusted HR 2.47, 95% CI 1.09–5.62). Patients with both impaired PET-derived MFR and diastolic dysfunction demonstrated more than five-fold increased risk of HFpEF hospitalization (*p* < 0.001). In another study, significant negative correlations were found between MFR and global circumferential strain (r = −0.29, *p* < 0.001), GLS (r = −0.33, *p* < 0.001), right ventricle longitudinal strain (r = −0.26, *p* < 0.001) and serum BNP levels (r = −0.32, *p* < 0.001) [34]. In addition, in our study, soluble ST2 was an independent factor associated with CMD in accordance with multivariate regression analysis data. Perhaps, in patients with non-obstructive CAD the levels of soluble ST2 reflect periarteriolar fibrosis that may occur in CMD [33]. Particularly, CMD related to chronic systemic inflammation may promote periarteriolar fibrosis and microvascular rarefaction, yielding decreased MFR and overexpression of this biomarker. In the study with mice models decreased ST2 signaling was connected with the progression of microvascular damage in the pressure overload state and myocardial periarteriolar fibrosis [33]. Thus, on the one hand, disturbances in both myocyte and non-myocyte compartments of the endothelium in chronic systemic inflammation cause adhesion and infiltration of monocytes and stimulation of integrated macrophages, which leads to differentiation of myofibroblasts and, ultimately, collagen secretion and, as a consequence, development of myocardial fibrosis, increased rigidity and diastolic dysfunction [4,7]. On the other hand, these pathophysiological mechanisms mediate the progression of hypoxia in tissues, which locally initiates the release of inflammatory cytokines [8]. Cytokines contribute to the formation of endothelial damage that might lead to perivascular fibrosis, thereby triggering a pathological circle of microcirculatory changes in the myocardium [8,9].

We also found an association of dynamic CZT-SPECT quantifications with parameters of diastolic dysfunction and GLS: MFR values correlated with LAVI, septal e′ and GLS, when rest-MBF correlated with E/e′. This suggests that factors tipping the balance towards cardiomyocyte injury in patients with existing CMD may deteriorate myocardial mechanics reflected in the reduction in GLS even in non-obstructive CAD [19]. In particular, CMD leads to decline nitric oxide bioavailability, and increased profibrotic cytokine signaling may contribute to reduce coronary microvascular rarefaction, and enlarged myocardial fibrosis [17]. Such interplay of disorders may synergize to propagate the vascular-ventricular stiffening in CMD thought to be central to the emerging epidemic of cardia disorders [25]. This theory has been also reflected in the MBF parameters. There is a distinction between patients with obstructive CAD who have decreased stress-MBF (due to flow limitation) and normal rest-MBF, and patients with CMD who have increased stress-MBF and decreased rest-MBF. On the one hand, in CMD patients, diffuse myocardial fibrosis leads to endothelium-dependent increasing in peripheral vascular resistance and increase in blood flow at rest. On the other hand, CMD associated with chronic systemic inflammation may promote periarteriolar fibrosis and microvascular rarefaction, yielding decreased stress-MBF [26]. Correlation between dynamic SPECT indices and biochemical markers of volume overload and diastolic function parameters has showed a closer relationship between these processes, and it has also particularly proved the pathogenesis of CMD [8,27]. Moreover, multivariate regression analysis also showed that the presence of diastolic dysfunction (OR 3.27; *p* < 0.001) and the hyperexpression of NT-proBNP ≥ 760.5 pg/mL (OR 1.67; *p* = 0.021) were independently associated with CMD. Our data do not contradict the results obtained in previous studies [7,17,29]. On the contrary, we have confirmed the fact that the presence of CMD is one of the grave triggers of the heart failure progression, ant the evidence of the relationship between the marker of hemodynamic overload NT-proBNP and CMD, as well as diastolic dysfunction, once again confirms the close relation between CMD and those processes.

## 5. Conclusions

Present results show for the first time that CMD obtained with dynamic CZT imaging is associated with the severe diastolic dysfunction, subtle LV systolic function impairment and is related to overexpression of the biomarkers of fibrosis and inflammation in low-risk patients with non-obstructive CAD and preserved LVEF. Presence of CMD in this population is interrelated with greater rate of adverse events and can individuate patients at high risk of adverse outcomes during the 12-month follow-up period. Despite our assessment of the integrity of endothelium-independent mechanisms of CMD in our study, these results could open the door to the development of preventive and therapeutic strategies that could improve the quality of life of affected patients and prognosis. Thus, further studies are needed to assess the role of endothelium-dependent mechanisms of CMD and to compare to other forms of ischemia with non-obstructive CAD. 

## 6. Limitation

The main limitations of the study were: (1) a relatively small and heterogeneous sample of patients, which does not exclude the possibility of the contribution of another pathology to the development of adverse outcomes; (2) the short follow-up period and small number of “hard” endpoints such death and hospitalizations.

We continue to observe patients and plan to have further adverse event results at long-term follow-up period. It is possible that over a longer follow-up period, patients will have not only endpoints associated with heart failure, but also MACE, which will allow us to confirm the contribution of CMD to the development of MACE in low-risk patients with preserved LVEF and non-obstructive CAD. Thus, further studies, with larger numbers of patients and longer follow-up, are needed to assess the role of CMD in the development of serious adverse cardiovascular events, and correlation with index of microcirculatory resistance, the invasive way to assess CMD, should be conducted to further validate the accuracy of the methodology to detect CMD.

## Figures and Tables

**Table 1:** Baseline clinical and demographic characteristics of patients.

Parameter	CMD+ *n* = 47	CMD− *n* = 73	*p*-Value
Age, years	61 (56; 68.5)	61.5 (59; 67.5)	0.123
Male sex, *n* (%)	26 (57.8)	44 (60.3)	0.901
Body mass index, kg/m^2^	29.9 (27.8; 31.9)	30.2 (27.9; 32.1)	0.276
Hypertension, *n* (%)	37 (82.2)	46 (63.0)	0.069
Type 2 diabetes mellitus, *n* (%)	11 (24.4)	6 (8.2)	0.007
History of COVID-19, *n* (%)	7 (15.6)	12 (16.4)	0.318
COPD, *n* (%)	7 (15.6)	13 (17.8)	0.723
Paroxysmal AF, *n* (%)	7 (15.6)	11 (15.1)	0.769
HFpEF, *n* (%)	34 (75.6)	24 (32.9)	<0.001
Smokers, *n* (%)	11 (24.4)	5 (6.8)	0.009
eGFR (mL/min/1.73 m^2^)	77.2 (63.2; 81.2)	77.0 (64.0; 85.0)	0.543
Total cholesterol, mmol/L	4.635 (3.67; 5.25)	4.33 (3.54; 4.98)	0.898
LDL-C, mmol/L	3.12 (2.15; 3.51)	2.87 (2.25; 3.87)	0.456
HDL-C, mmol/L	1.05 (0.83; 1.32)	1.05 (0.96; 1.26)	0.887
Triglyceride, mmol/L	1.67 (1.23; 1.89)	1.59 (1.22; 1.86)	0.835
Hemoglobin, g/dL	134 (121; 143)	137 (128; 142)	0.464
Potassium, mmol/L	4.64 (4.12; 5.01)	4.81 (4.43; 5.21)	0.517
Fibrinogen, g/L	3.27 (3.14; 3.14)	3.10 (2.86; 3.43)	0.767
HbA1c, %	5.9 (5.1; 6.9)	5.8 (5.1; 6.4)	0.098
β-blockers, *n* (%)	8 (17.8)	10 (13.7)	0.876
ACE inhibitors/ARBs, *n* (%)	5 (11.1)	9 (12.0)	0.879
Diuretics, *n* (%)	3 (6.7)	8 (10.9)	0.546
Statins, *n* (%)	8 (17.8)	15 (20.5)	0.547
Amiodarone, *n* (%)	2 (4.4)	5 (6.8)	0.358
ARA, *n* (%)	2 (4.4)	4 (5.5)	0.269

Note. CMD—Coronary microvascular dysfunction; COVID-19—Coronavirus disease 2019; COPD—Chronic obstructive pulmonary disease; HFpEF—Heart failure with preserved ejection fraction; eGFR—Estimated glomerular filtration rate; LDL-C—Low-Density Lipoprotein Cholesterol; HDL-C—High-Density Lipoprotein Cholesterol; HbA1c—Glycated hemoglobin; ACE—Angiotensin-converting enzyme inhibitors; ARBs—Angiotensin II receptor blockers; ARA—Aldosterone receptor antagonists.

**Table 2:** Baseline echocardiographic and dynamic SPECT parameters.

Parameter	CMD+ *n* = 47	CMD− *n* = 73	*p*-Value
Echocardiographic parametrs			
Left ventricle ejection fraction, %	62 (58.5; 65.0)	63 (61; 66)	0.183
End-systolic dimension, mm	40 (38; 43)	38.5 (36.5; 41.5)	0.524
End-diastolic dimension, mm	51.0 (48.7; 53.0)	50.5 (47.5; 52.5)	0.307
LVMMi, g/m^2^	98.0 (88.5; 114.5)	92 (85.5; 106.5)	0.276
E/A ratio	1.04 (0.79; 1.3)	0.97 (0.74; 1.2)	0.516
Lateral e′, sm/s	5.56 (4.78; 6.45)	8.56 (8.01; 9.14)	0.009
TRV, m/s	2.99 (2.95; 3.01)	2.63 (2.3; 2.76)	0.011
E/e′ ratio	14 (13.5; 15.0)	11 (10; 12)	0.041
LAVI, mL/m^2^	38.3 (35.7; 51.1)	29.7 (27.5; 47.9)	0.038
LV global longitudinal strain, %	−14.7 (−12.9; −16.9)	−20.9 (16.1; 21.6)	0.005
Diastolic dysfunction, *n* (%)	37 (88.1)	26 (37.1)	<0.001
Dynamic SPECT parametrs			
Stress-MBF, mL/min/g	1.14 (0.67; 1.49)	1.63 (1.19; 1.83)	<0.001
Rest-MBF, mL/min/g	0.75 (0.54; 0.99)	0.52 (0.40; 0.69)	<0.001
Myocardial flow reserve	1.39 (1.11; 1.96)	2.69 (2.15; 3.78)	<0.001
Standard semi-quantitative indices of myocardial perfusion imaging			
Summed stress score	3 (0.5; 4)	2.5 (0; 5)	0.753
Summed rest scores	2 (0; 3)	1 (0; 2)	0.537
Difference between stress and rest score	2 (0; 3)	2 (0; 4)	0.975

Note. E/A—the ratio of the maximum blood flow rate in the phase of rapid filling to the maximum flow rate in atrial systole; E/e′—the ratio of the transmitral E peak to the tissue myocardial Doppler e′; LVMMi—left ventricular myocardial mass index; LAVI—left atrial volume indexed; TRV—tricuspid regurgitation peak velocity; lateral e′—early diastolic velocity of the lateral wall of the left ventricle.

**Table 3:** The baseline levels of biomarkers.

**Parameter**	**CMD+** ***n* = 47**	**CMD−** ***n* = 73**	***p*-Value**
NT-proBNP, pg/mL	404.2 (249.5; 1533.4)	156.3 (135.26; 274.7)	0.004
IL-10, pg/mL (N < 10 pg/mL)	2.87 (2.58; 3.57)	3.67 (3.32; 4.04)	0.048
IL-1β, pg/mL (N < 11 pg/mL)	3.19 (1.64; 5.47)	1.2 (0.74; 1.48)	0.046
IL-6, pg/mL (N < 31 pg/mL)	2.65 (1.98; 3.98)	2.48 (1.87; 3.76)	0.842
hsCRP, g/L (N < 12 g/L)	4.1 (3.0; 11.4)	2.3 (1.1; 8.7)	0.009
Soluble ST2, ng/mL	33.67 (27.65; 38.9)	27.5 (21.78; 30.09)	<0.001
TIMP-1, ng/mL	287.4 (107.38; 371.8)	123.64 (58.66; 232.9)	0.011
MMP-9, ng/mL	2109 (1145.7; 3235)	1104 (721.5; 1731.9)	0.012
Tetranectin, ng/mL	6.83 (6.31; 7.68)	7.03 (6.29; 7.82)	0.786
FGF-23, ng/mL	0.691 (0.465; 1.042)	0.672 (0.509; 0.976)	0.567

Note. NT-proBNP—N-terminal pro-B-type natriuretic peptide; IL—interleukin; hsCRP—high-sensitivity C-reactive protein; TIMP-1—tissue inhibitor of metalloproteinase-1; MMP-9—matrix metalloproteinase 9; FGF-23—fibroblast growth factor 23.

**Table 4:** Results of univariate and multivariate regression analysis.

Parameter	Odds Ration	95% CI	*p*-Value
Univariate regression analysis			
Type 2 diabetes mellitus	1.43	1.17–3.57	0.012
NT-proBNP (<760.5/≥760.5 pg/mL)	2.13	1.78–4.87	0.009
hsCRP (<3.2/≥3.2 g/L)	1.63	0.98–2.54	0.013
Diastolic dysfunction	3.18	1.16–4.12	<0.001
Smoking	2.01	0.99–2.43	0.043
Soluble ST2 (<31.4/≥31.4 ng/mL)	1.97	1.16–5.12	0.003
Multivariate regression analysis			
Diastolic dysfunction	3.27	2.26–5.64	<0.001
NT-proBNP (<760.5/≥760.5 pg/mL)	1.67	1.12–4.15	0.021
Soluble ST2 (<31.4/≥31.4 ng/mL)	1.33	1.08–3.19	0.025

Note. CI—confidential interval; NT-proBNP—N-terminal pro-B-type natriuretic peptide; hsCRP—high-sensitivity C-reactive protein.

## Data Availability

The data presented in this study are available on request from the corresponding author. The data are not publicly available due to privacy.

## References

[1] Masi S., Rizzoni D., Taddei S., Widmer R.J., Montezano A.C., Lüscher T.F., Schiffrin E.L., Touyz R.M., Paneni F., Lerman A. (2021). Assessment and pathophysiology of microvascular disease: Recent progress and clinical implications. Eur. Heart J..

[2] Houben A.J., Stehouwer C.D. (2021). Microvascular dysfunction: Determinants and treatment, with a focus on hyperglycemia. Endocr. Metab. Sci..

[3] Godo S., Suda A., Takahashi J., Yasuda S., Shimokawa H. (2021). Coronary Microvascular Dysfunction. Arterioscler. Thromb. Vasc. Biol..

[4] Likoff W., Segal B.L., Kasparian H. (1967). Paradox of normal selective coronary arteriograms in patients considered to have unmistakable coronary heart disease. N. Engl. J. Med..

[5] Borbély A., van der Velden J., Papp Z., Bronzwaer J.G.F., Edes I., Stienen G.J.M., Paulus W.J. (2005). Cardiomyocyte stiffness in diastolic heart failure. Circulation.

[6] Murthy V.L., Naya M., Taqueti V.R., Foster C.R., Gaber M., Hainer J., Dorbala S., Blankstein R., Rimoldi O., Camici P.G. (2014). Effects of sex on coronary microvascular dysfunction and cardiac outcomes. Circulation.

[7] Mileva N., Nagumo S., Mizukami T., Sonck J., Berry C., Gallinoro E., Monizzi G., Candreva A., Munhoz D., Vassilev D. (2022). Prevalence of Coronary Microvascular Disease and Coronary Vasospasm in Patients with Nonobstructive Coronary Artery Disease: Systematic Review and Meta-Analysis. J. Am. Heart Assoc..

[8] Rush C.J., Berry C., Oldroyd K.G., Rocchiccioli J.P., Lindsay M.M., Touyz R.M., Murphy C.L., Ford T.J., Sidik N., McEntegart M.B. (2021). Prevalence of Coronary Artery Disease and Coronary Microvascular Dysfunction in Patients with Heart Failure With Preserved Ejection Fraction. JAMA Cardiol..

[9] Shah S.J., Lam C.S.P., Svedlund S., Saraste A., Hage C., Tan R., Beussink-Nelson L., Faxén U.L., Fermer M.L., Broberg M.A. (2018). Prevalence and correlates of coronary microvascular dysfunction in heart failure with preserved ejection fraction: PROMIS-HFpEF. Eur. Heart J..

[10] Liga R., Neglia D., Kusch A., Favilli B., Giorgetti A., Gimelli A. (2022). Prognostic Role of Dynamic CZT Imaging in CAD Patients: Interaction Between Absolute Flow and CAD Burden. JACC Cardiovasc. Imaging.

[11] Kato S., Saito N., Kirigaya H., Gyotoku D., Iinuma N., Kusakawa Y., Iguchi K., Nakachi T., Fukui K., Futaki M. (2016). Impairment of coronary flow reserve evaluated by phase contrast cine-magnetic resonance imaging in patients with heart failure with preserved ejection fraction. J. Am. Heart Assoc..

[12] Mohammed S.F., Hussain S., Mirzoyev S.A., Edwards W.D., Maleszewski J.J., Redfield M.M. (2015). Coronary microvascular rarefaction and myocardial fibrosis in heart failure with preserved ejection fraction. Circulation.

[13] Taqueti V.R., Solomon S.D., Shah A.M., Desai A.S., Groarke J.D., Osborne M.T., Hainer J., Bibbo C.F., Dorbala S., Blankstein R. (2018). Coronary microvascular dysfunction and future risk of heart failure with preserved ejection fraction. Eur. Heart J..

[14] Yang J.H., Obokata M., Reddy Y.N., Redfield M.M., Lerman A., Borlaug B.A. (2020). Endothelium-dependent and independent coronary microvascular dysfunction in patients with heart failure with preserved ejection fraction. Eur. J. Heart Fail..

[15] Zavadovsky K.V., Mochula A.V., Boshchenko A.A., Vrublevsky A.V., Baev A.E., Krylov A.L., Gulya M.O., Nesterov E.A., Liga R., Gimelli A. (2021). Absolute myocardial blood flows derived by dynamic CZT scan vs invasive fractional flow reserve: Correlation and accuracy. J. Nucl. Cardiol..

[16] Henzlova M.J., Duvall W.L., Einstein A.J., Travin M.I., Verberne H.J. (2016). ASNC imaging guidelines for SPECT nuclear cardiology procedures: Stress, protocols, and tracers. J. Nucl. Cardiol..

[17] Cerqueira M.D., Weissman N.J., Dilsizian V., Jacobs A.K., Kaul S., Laskey W.K., Pennell D.J., Rumberger J.A., Ryan T., Verani M.S. (2002). Standardized Myocardial Segmentation and Nomenclature for Tomographic Imaging of the Heart: A Statement for Healthcare Professionals from the Cardiac Imaging. Circulation.

[18] Nagueh S.F., Smiseth O.A., Appleton C.P. (2016). Recommendations for the evaluation of left ventricular diastolic function by echocardiography: An update from the American Society of Echocardiography and the European Association of Cardiovascular Imaging. Eur. Heart J. Cardiovasc. Imaging.

[19] Jovanovic I., Tesic M., Giga V., Dobric M., Boskovic N., Vratonjic J., Orlic D., Gudelj O., Tomasevic M., Dikic M. (2020). Impairment of coronary flow velocity reserve and global longitudinal strain in women with cardiac syndrome X and slow coronary flow. J. Cardiol..

[20] Voigt J.-U., Pedrizzetti G., Lysyansky P., Marwick T.H., Houle H., Baumann R., Pedri S., Ito Y., Abe Y., Metz S. (2015). Definitions for a common standard for 2D speckle tracking echocardiography: Consensus document of the EACVI/ASE/Industry Task Force to standardize deformation imaging. Eur. Heart J. Cardiovasc. Imaging.

[21] McDonagh T.A., Metra M., Adamo M., Gardner R.S., Baumbach A., Böhm M., Burri H., Butler J., Čelutkienė J., Chioncel O. (2021). 2021 ESC Guidelines for the diagnosis and treatment of acute and chronic heart failure. Eur. Heart J..

[22] Weerts J., Mourmans S.G.J., Aizpurua A.B., Schroen B.L.M., Knackstedt C., Eringa E., Houben A.J.H.M., van Empel V.P.M. (2022). The Role of Systemic Microvascular Dysfunction in Heart Failure with Preserved Ejection Fraction. Biomolecules.

[23] Obokata M., Reddy Y.N., Melenovsky V., Kane G.C., Olson T.P., Jarolim P., Borlaug B.A. (2018). Myocardial injury and cardiac reserve in patients with heart failure and preserved ejection fraction. J. Am. Coll. Cardiol..

[24] Ahmad A., Corban M.T., Toya T., Verbrugge F.H., Sara J.D., Lerman L.O., Borlaug B.A., Lerman A. (2021). Coronary microvascular dysfunction is associated with exertional haemodynamic abnormalities in patients with heart failure with preserved ejection fraction. Eur. J. Heart Fail..

[25] Sinha A., Rahman H., Webb A., Shah A.M., Perera D. (2021). Untangling the pathophysiologic link between coronary microvascular dysfunction and heart failure with preserved ejection fraction. Eur. Heart J..

[26] Boden W.E., O’Rourke R.A., Teo K.K., Hartigan P.M., Maron D.J., Kostuk W.J., Knudtson M., Dada M., Casperson P., Harris C.L. (2007). Optimal medical therapy with or without PCI for stable coronary disease. N. Engl. J. Med..

[27] Maron D.J., Hochman J.S., Reynolds H.R., Bangalore S., O’Brien S.M., Boden W.E., Chaitman B.R., Senior R., López-Sendón J., Alexander K.P. (2020). Initial invasive or conservative strategy for stable coronary disease. N. Engl. J. Med..

[28] Shimokawa H., Suda A., Takahashi J., Berry C., Camici P.G., Crea F., Escaned J., Ford T., Yii E., Kaski J.C. (2021). Clinical characteristics and prognosis of patients with microvascular angina: An international and prospective cohort study by the Coronary Vasomotor Disorders International Study (COVADIS) Group. Eur. Heart J..

[29] Schroder J., Michelsen M.M., Mygind N.D., E Suhrs H., Bove K.B., Bechsgaard D.F., Aziz A., Gustafsson I., Kastrup J., Prescott E. (2021). Coronary flow velocity reserve predicts adverse prognosis in women with angina and no obstructive coronary artery disease: Results from the iPOWER study. Eur. Heart J..

[30] Kato S., Fukui K., Kodama S., Azuma M., Nakayama N., Iwasawa T., Kimura K., Tamura K., Utsunomiya D. (2021). Cardiovascular magnetic resonance assessment of coronary flow reserve improves risk stratification in heart failure with preserved ejection fraction. J. Cardiovasc. Magn. Reson..

[31] Gallinoro E., Paolisso P., Candreva A., Bermpeis K., Fabbricatore D., Esposito G., Bertolone D., Fernandez Peregrina E., Munhoz D., Mileva N. (2021). Microvascular Dysfunction in Patients with Type II Diabetes Mellitus: Invasive Assessment of Absolute Coronary Blood Flow and Microvascular Resistance Reserve. Front. Cardiovasc. Med..

[32] Crea F., Merz C.N.B., Beltrame J.F., Kaski J.C., Ogawa H., Ong P., Sechtem U., Shimokawa H., Camici P.G. (2017). The parallel tales of microvascular angina and heart failure with preserved ejection fraction: A paradigm shift. Eur. Heart J..

[33] Filali Y., Kesäniemi A., Ukkola O. (2021). Soluble ST2, a biomarker of fibrosis, is associated with multiple risk factors, chronic diseases and total mortality in the OPERA study. Scand. J. Clin. Lab. Investig..

[34] Rehan R., Yong A., Ng M., Weaver J., Puranik R. (2023). Coronary microvascular dysfunction: A review of recent progress and clinical implications. Front. Cardiovasc. Med..

